# A Continuous Identity Authentication Scheme Based on Physiological and Behavioral Characteristics

**DOI:** 10.3390/s18010179

**Published:** 2018-01-10

**Authors:** Guannan Wu, Jian Wang, Yongrong Zhang, Shuai Jiang

**Affiliations:** School of Electronic Science, National University of Defense Technology, Changsha 410073, China; wuguannan12@nudt.edu.cn (G.W.); zhangyongrong09@nudt.edu.cn (Y.Z.); jiangshuai11@nudt.edu.cn (S.J.)

**Keywords:** identity authentication, wearable device, multisensor data, human activity recognition, machine learning algorithm

## Abstract

Wearable devices have flourished over the past ten years providing great advantages to people and, recently, they have also been used for identity authentication. Most of the authentication methods adopt a one-time authentication manner which cannot provide continuous certification. To address this issue, we present a two-step authentication method based on an own-built fingertip sensor device which can capture motion data (e.g., acceleration and angular velocity) and physiological data (e.g., a photoplethysmography (PPG) signal) simultaneously. When the device is worn on the user’s fingertip, it will automatically recognize whether the wearer is a legitimate user or not. More specifically, multisensor data is collected and analyzed to extract representative and intensive features. Then, human activity recognition is applied as the first step to enhance the practicability of the authentication system. After correctly discriminating the motion state, a one-class machine learning algorithm is applied for identity authentication as the second step. When a user wears the device, the authentication process is carried on automatically at set intervals. Analyses were conducted using data from 40 individuals across various operational scenarios. Extensive experiments were executed to examine the effectiveness of the proposed approach, which achieved an average accuracy rate of 98.5% and an F1-score of 86.67%. Our results suggest that the proposed scheme provides a feasible and practical solution for authentication.

## 1. Introduction

The development of smart devices is undeniably transforming the way of our daily life. Recent surveys [1,2] show the great potential of loT (Internet of Things) technology (e.g., smart appliances, wearable devices, and home automation). However, these applications also present potential risks like unauthorized access. The most common mechanism to address the unauthorized access issue is the authentication. Authentication methods include PIN (Personal Identification Number) passcodes, smart cards, and biometrics (e.g., fingerprints, face recognition, and gait recognition). However, most passcodes are either simply decoded or require intentional memory [3,4]. Some studies also showed that by using the embedded motion sensor, one can infer a user’s input number for a smartphone [5,6]. Smart cards require auxiliary hardware and may cause loss problem. Additionally, they suffer from security attacks, including power analysis attack [7] and fault injection [8]. Biometrics are influenced by the environment. For instance, fingerprint recognition is influenced by the humidity and molting of human fingers. Also, some experiments showed that fingerprints could be counterfeited by using putty and gelatin or a high-quality scanner [9]. Face recognition is affected by light and shelter. Also, a facial recognition system has to fight against spoof attacks that a photo of a legitimate user may obtain access to the system. In addition, most authentication methods adopt a one-time manner. Once an illegal user gains access, he could be regarded as a legal user without recertification for a long period of time.

The maturity of sensor chip integration, artificial intelligence, and machine learning algorithms provide us with an effective solution for identification and authentication. Miniature sensors can be unobtrusively attached to the body to discern and attest a user’s ID. In this paper, we propose an applicable study on continuous authentication based on an own-built fingertip multisensor device. In order to achieve a high-precision, continuous authentication, we used a device to collect motion data and physiological data recognizing the state of motion. After correctly inferring the movement, a one-class machine learning algorithm was applied for user authentication. In our experiments, three light-weight classifiers were applied for activity recognition and three one-class classification algorithms were employed to achieve the authentication task. We tested three common motions for activity recognition, namely, stationary state, slow walking state, and strenuous movements. In addition, we also examined the performance on usability with respect to window size and training sample size. In the end, we propose a reliable and practical authentication scheme. The results show a better performance of the authentication method based on both physiological and behavioral characteristics compared to one-time methods based on a single authentication parameter.

This paper is organized as follows. Section 2 introduces the background and related works; Section 3 presents the system block diagram, illustrates the application scenarios, and addresses the data collection process and related apparatus; Section 4 explains the details of the multisensor data analysis, feature selection, and classifier implementation; Section 5 presents the experimental results and provides an extensive analysis of the experiments; Section 6 summarizes the main work of this paper and highlights future work.

## 2. Background and Related Work

### 2.1. Identity Authentication Based on Motion Sensors

Currently, the application of motion sensor is mainly for Human Activity Recognition (HAR). Janidarmian et al. [10] presented a comprehensive analysis on a wearable acceleration sensor for HAR. They examined the accuracy performance with respect to common machine learning algorithms and versatility with respect to 14 well-known benchmark datasets and different types of acceleration sensors. Also, deep learning algorithms were applied for HAR and proved to be highly precise [11,12,13].

Research on authentication by motion sensors is relatively new. Recently, with the increasing capability of smartphones, Ehatishamulhaq et al. [14] used the embedded motion sensors of smartphone for users’ authentication. They applied several classifiers to recognize different activities, then authenticated the identity of a user based on the prior knowledge of their motion states. The experiments showed an authentication accuracy rate of 91.67%. Shen et al. [15] used smartphone accelerometers and orientation sensors to authenticate a user through the action of passcode input. They utilized different levels of the user’s posture and of the smartphone changes of motion as distinctive characteristics. By employing three common machine learning algorithms (SVM, Supported Vector Machine), Neural Network, and Nearest-Neighbor), the experiments showed a false rejection rate (FRR) of 6.85% and a false acceptance rate (FAR) of 5.01%. Conti et al. [16] used a similar method and exploited the differences in the way of answering the phone. Through the process of Dynamic Time Warping (DTW), their experiments showed an impostor pass rate of 4.5% and a false acceptance rate of 9.5%. However, most smartphone authentication methods belong to the static authentication type and check the user’s identity only once at login. In addition, some work did not examine the differences in the raw data when the same user performed the same movement in different periods of time, which may introduce variations.

The works mentioned above showed the application of smartphone built-in motion sensors to achieve user authentication. Also, some studies focused on body-worn motion sensors to achieve authentication. Xu et al. [17] proposed a face recognition method for smart glass based on both camera and Inertial Measurement Unit (IMU) sensors. They utilized the angle information collected from IMU sensors to improve the recognition accuracy. The results showed an improvement of accuracy of 15% under the same computation cost of other methods. Zhang et al. [18] used gait as the biometric for identity recognition. To avoid the cycle detection failure, a novel multiscale Signature Points (SP) extraction method was proposed for recognition. The recognition rate reached 95.8% by using five accelerometers on different body locations. Zeng et al. [19] shared similar thoughts with us. They investigated the possibility of using dynamic behavior as a unique marker of users to design an implicit authentication framework for wearable devices. The rationale behind their work is the unique pattern of every user when they perform specific activities. Firstly, they built an activity classifier to distinguish four simple activities, namely, walking, running, climbing, and jumping, and then, they built an activity-specific authentication model. Also, different placements of the motion sensor were considered. The experiments showed the lowest false-positive rate of 0.3% when the walking state was detected correctly. Cola et al. [20] used motion data collected from a user’s wrist for authentication. A detection algorithm was proposed to discern whether a user was walking or not, and an anomaly detection method was used to distinguish genuine inputs from unauthorized users. Their results showed an equal error rate (EER) of 2.9% in an experiment with 15 volunteers. Xu et al. [21] reduced the power consumption of an accelerometer by utilizing the output voltage signal from a kinetic energy harvester (KEH). They used the unique patterns from wearable KEH devices for authentication. The results showed that the power consumption was reduced by 78.5% while the accuracy was 6% lower.

We can infer that most gait authentication schemes provided a continuous authentication solution which verified the identity unobtrusively throughout the usage period. However, most of the works required that the user remained in a kinetic state, which could not provide authentication when the user was in a stationary state.

### 2.2. Identity Authentication Based on Physiological Sensors

Because of the unique and hard-forged characteristics of human biology, biometrics has emerged as a novel and robust technology in many verification tasks. At present, biometric verification methods based on fingerprint [22], face [23], and voice [24] have been used widely and proved to have relatively high accuracy. Yet, recent study showed that the above methods forged security risks [9,25]. Scientists have proposed to use other unique characteristics like, among others, ear [26], vein [27], odor [28], electroencephalograph (EEG) [29], electrocardiogram (ECG) [30], and photoplethysmography (PPG) [31]. However, some biometrics, such as vein, odor, and EEG, are hard to integrate into wearable devices. Since this paper is mainly focused on wearable sensors, we will mainly describe body-worn biometric devices.

Nakanishi et al. [32] verified the authentication performance of intrabody propagation signals. However, the accuracy was low because of the influence of white noise. Li et al. [33] used the transmission gain S21 as a biometric trait for personal verification. The emission electrode and receiving electrode were placed on a volunteer’s forearm. They also proposed a threshold adaptive template-matching method based on Euclidean distance which achieved a false acceptance rate of 5.79% and a false rejection rate of 6.74%. However, they tested only in the laboratory without considering the interference of external electromagnetic signals.

Usually, users have to carry a bulky instrument for continuous ECG monitoring. With the rapid development of microsensors and microprocessors, a small-size, compact wearable ECG sensor were made recently [34]. Camara et al. [35] utilized ECG signals for user identification. A k-NN algorithm was applied after non-fiducial feature extraction via Hadamard Transform. The experiments showed an accuracy rate of 97% and an error rate around 10%. A similar idea was presented in [36]. The researchers performed a multi-class SVM by using kernel function after Discrete Wavelet Transform (DWT). The results showed a false match rate around 3.97%. Although these works have shown a great potential for ECG-based authentication, the prerequisite was that the wearer remained stationary, under low levels of environmental noise. Kang et al. [37] collected the ECG signal through wearable watches and experimented under high levels of noise. The results showed a false acceptance rate of 5.2% and a false rejection rate of 1.9%. However, they kept the user in a particular state of motion, which limited the practical application. These efforts described above showed that the ECG sensors have a rich potential for user authentication. However, the ECG equipment still needs to be attached to the chest or to both hands, which is not convenient when the user is moving.

The utilization of PPG signals has also been proved to be a novel way for biometric authentication. Da et al. [38] placed a PPG signal collector on the fingertip of the subject and applied DTW for measuring the similarity between the sample and a template. The results showed a FAR of 2% and a FRR of 10% under the optimal threshold. Spachos et al. [39] applied the Linear Discriminant Analysis as an extraction tool and the Nearest-Neighbor as a classifier. The results showed a FAR of 5% and a FRR of 30%. However, the subjects were required to remain static and breath evenly, which is unrealistic. Ohtuski et al. [40] used a wrist-worn PPG sensor to measure the waveform of different wrist movements, like flicking. They then extracted nine time-domain features for Random Forest Classifier. Yet, the user was required to stay still for five seconds, which was obtrusive, and the wrist-worn PPG sensor could not acquire accurate raw data.

As described above, single-sensor certifications cannot provide yet high accuracy and extensive applicability simultaneously. To our knowledge, few papers have addressed the analysis of continuous authentication using data from both kinetic sensors and biometric sensors. Miao et al. [41] provided a wearable ECG monitoring system integrated with the built-in motion sensors of a smartphone. They installed the ECG acquisition device on the user’s chest to get ECG raw data and transmit it to a smartphone via Bluetooth. Meanwhile, the smartphone applied the activity recognition method based on the built-in motion sensors. This system could analyze ECG abnormal patterns with a prior knowledge of the motion state. However, it was used in medical diagnosis instead of authentication, and, in addition, the ECG device installed on the chest was uncomfortable for the user. Similarly, Kos et al. [42] recorded the data from an inertial sensor and a PPG sensor to detect and examine tennis gestures for training purposes. The device was portable and easily wearable, yet their research included just raw data analysis and only applied some simple feature extraction methods.

In general, wearable sensors have shown a great potential for identity authentication, yet there are still several problems and deficiencies, such as low precision, discomfort of wearing a sensor, and application restrictions. Thus, we combined motion sensors and a physiological sensor to achieve high-precision authentication. The motion sensors included an accelerometer and a gyroscope, and the physiological sensor included a PPG sensor. We chose PPG instead of ECG because it could supply measures through a single finger. Because the PPG signal is affected by body movements, the system firstly applies an activity recognition method to detect whether the wearer is doing relatively strenuous movements or not, then it authenticates the user under slow motion and stationary state. Compared to the existing works, this study: (1) aims to provide a high-precision authentication system using both motion sensors (accelerometer and gyroscope) and a physiological sensor (PPG signal); (2) achieves continuous authentication with a small-size, light fingertip device which collects raw data automatically; (3) employs a low computational classification algorithm for activity recognition. We considered three categories for classification: slowly walking, sitting, and strenuous movements, including trotting and ascending and descending stairs; (4) employs a one-class classifier to build the authentication model. More specifically, we used PPG and motion data to accomplish authentication during a slow walking state and used PPG solely during a stationary state; (5) examines a set of classifiers both for activity recognition and for identity authentication.

## 3. The Proposed Multisensor Data Authentication System 

This section explains how we designed the multisensor authentication system and specifies the application scenario of our method. We also give details of the related sensor device and the process of sample data acquisition.

### 3.1. System Architecture and Application Scenario

In this subsection, an example scenario is illustrated to introduce the procedure and application of the proposed system.

Example Scenario. Companies usually have archive centers to place important documents. Users need to gain entrance authority from authentication equipment like fingerprint and face recognition devices, or by simply using a key. In order to further improve security, users need to wear the fingertip device and walk a certain distance. The fingertip device will authenticate the identity under the walking state. Most authentication systems only require a one-time authentication when the user attempts to walk into the room. Once an illegal user gains access, he could be regarded as a legal user without recertification for a long period of time. Our fingertip device addresses this issue. Normally, users will browse some paper documents or perform other slow motions when they get into the archive. The fingertip device will authenticate the identity under the stationary state. Also, it is considered an act of interference by an illegal user if the system continues to detect a strenuous state.

**Figure 1:**
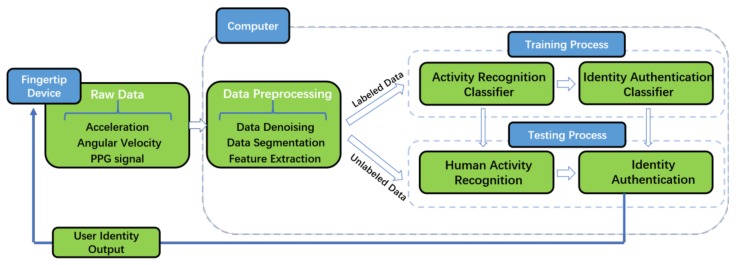
System architecture of our multisensor authentication scheme.

The block diagram of the proposed multisensor authentication scheme with a fingertip device is described in Figure 1. A user wears the sensor device on the fingertip and the device will automatically obtain data including acceleration, angular velocity, magnetic intensity, and PPG signals (we did not use magnetometer data). The Arduino Uno platform receives the raw data and transmits it to a computer through Bluetooth for further analysis. Data preprocessing is then conducted to denoise the raw data and extract representative features. In the training phase, the computer labels the raw data with three types of motion states (slow walking, sitting, and strenuous movements), and then applies the classifier with less computational complexity for activity recognition. Moreover, the authentication classifiers are constructed by training walking labeled data and sitting labeled data, respectively. In the testing phase, the motion state is detected based on the activity recognition classifier. Then, an activity specific authentication classifier is applied. Once the authentication is done, the computer will send an authentication command back to fingertip device to evaluate whether the wearer is a legal user. The testing phase is automatic and unobtrusive.

### 3.2. Apparatus

**Figure 2:**
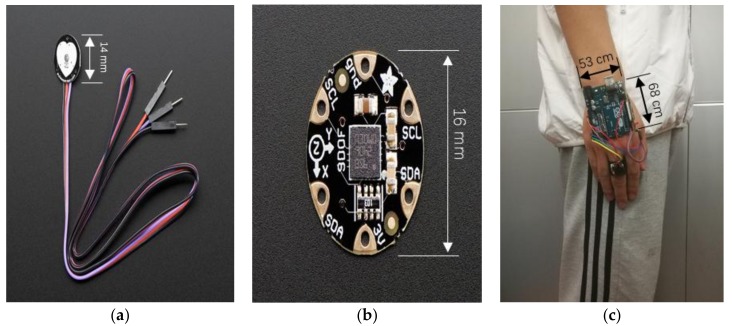
Photography of the sensors and the device: (**a**) Pulse Sensor Amped, (**b**) FLORA 9-DOF LSM9DS0, and (**c**) our fingertip device.

We established a small-size and portable sensor device based on two sensor chips, as shown in Figure 2, FLORA 9-DOF LSM9DS0 and Pulse Sensor Amped. The FLORA project allowed us to detect motion, direction, and orientation through high-precision 9-DOF LSM9DS0 sensors, including a 3-axis accelerometer, a 3-axis gyroscope, and a 3-axis magnetometer. The diameter and thickness of the chip were 16 mm and 0.8 mm, respectively. We set the accelerometer range to ±4g, where “g” denotes the gravitational acceleration, and set 500 DPS (Degree Per Second) to the gyroscope. The Pulse Sensor Amped is essentially a photoplethysmogram which measures the blood oxygen levels. It responds to relative changes in green LED light intensity which generate the pulse wave. The device also amplifies the raw signal of the pulse and normalizes the wave after filtering. All the sensors were initially time-synchronized and collected data at a fixed sampling rate of 50 Hz. We used the Arduino Uno for data transmission with the baud rate of 9600 bit/s.

### 3.3. Data Collection Process

We recruited 40 volunteers to perform the task (30 males and 10 females). Each user was required to perform three groups of actions for 20 repetitions, each day. Each repetition lasted around 12 s, so we had $\underset{\mathrm{sampling}\;\mathrm{rate}}{\underset{︸}{50}}\times \underset{\mathrm{sampling}\;\mathrm{time}}{\underset{︸}{12}}=600$ samples for each case. These actions included slow walking, sitting, and doing relatively strenuous movements, like trotting and ascending and descending stairs. The sensor device which was fastened on the fingertip collected the raw data and transmitted it to a computer through Bluetooth for further analysis. All 40 subjects were asked to perform the experiments under their natural conditions (the portable sensor device ensures that the users feel comfortable). Considering the impact of time-to-time variation on behavior habits, we performed the experiments at different times during 30 days. The final dataset contains $\underset{\begin{array}{l} \mathrm{volunteer}\\ \;\mathrm{number} \end{array}}{\underset{︸}{40}}\times \underset{\begin{array}{l} \;\mathrm{day}\\ \mathrm{number} \end{array}}{\underset{︸}{30}}\times \underset{\begin{array}{l} \;\mathrm{repetitions}\;\mathrm{every}\;\mathrm{group}\\ \;\mathrm{per}\;\mathrm{day}\;\mathrm{per}\;\mathrm{user} \end{array}}{\underset{︸}{20}}\times \underset{\begin{array}{l} \;\mathrm{group}\\ \mathrm{number} \end{array}}{\underset{︸}{3}}=72,000$ cases. Our dataset is available [43].

## 4. Fusion Sensor Data Analysis

This section presents the details about the preprocessing of raw data, feature extraction, and classifier implementation.

### 4.1. Data Denoising and Segmentation

**Figure 3:**
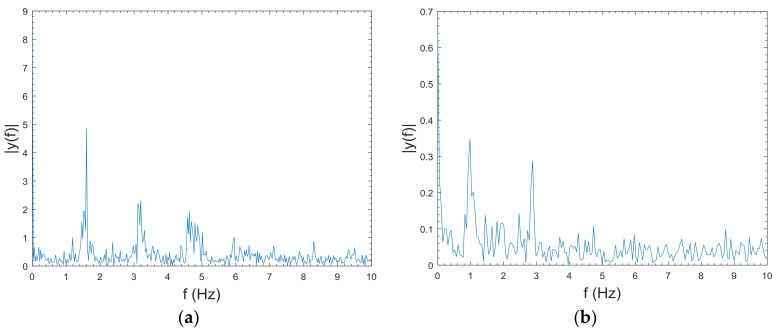
Single-sided amplitude spectrum of sensor data: (**a**) PPG signal, and (**b**) acceleration signal.

The sensor signals are sensitive to disturbances such as power interference and white noise. Figure 3 shows the frequency spectrogram of the PPG signal and the acceleration signal when the user was trotting. The PPG signal spans frequencies between 0 Hz to 5 Hz, and the acceleration signal between 0 Hz to 3 Hz. To mitigate the effect of noises that are not intrinsic to the data, filter methods should be employed. Filters like Kalman filter, Weiner filter, and Adaptive filter are all great filters, yet they require information on the desired signal and a certain calculation cost. Considering the trade-off between computing complexity and denoising performance, a fourth-order Chebyshev low-pass filter with a cutoff frequency of 5 Hz was applied to reduce the noise. Also, we used the same filter under walking and stationary states, because the movements under these states contain components at a lower frequency than the cutoff frequency.

The original sequence usually contains multiple similar motion periods. Thus, data segmentation is necessary to reduce the computation cost. Banos et al. [44] showed that window size could crucially impact the activity recognition process. To address this challenge, we applied the Sliding Window method. Fixed-size Sliding Window (FSW) is the most common and easiest way, where data sequence is segmented into fixed-length subsequences. In our work, we analyzed the influence of window sizes ranging from 2 s to 12 s with the degree of overlapping set as 20%.

**Figure 4:**
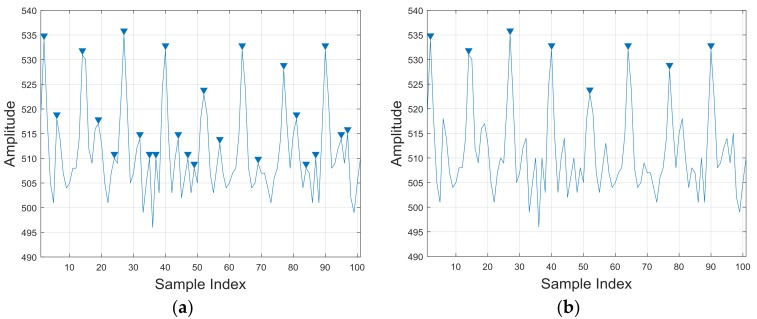
Cycle detection of PPG signal: (**a**) first-order derivative method and (**b**) our method.

In order to extract the periodicity of PPG signals as a feature, we applied a peak detection method. The calculation of the first derivative of signal is the most immediate way to find peaks, yet the signal may suffer from high-frequency noise which causes pseudo-maximum points. In this paper, we measured the distance between peaks in the sequence to detect cycles. However, because of the nonstationary noise, some of the peaks could seem very close to each other. We considered that peaks should satisfy a drop-off on both sides by eight sample data width. Figure 4 shows the results obtained by the derivative way and our way. It is clearly shown that our method proved to perform better.

### 4.2. Activity Recognition Method

#### 4.2.1. Feature Extraction

**Figure 5:**
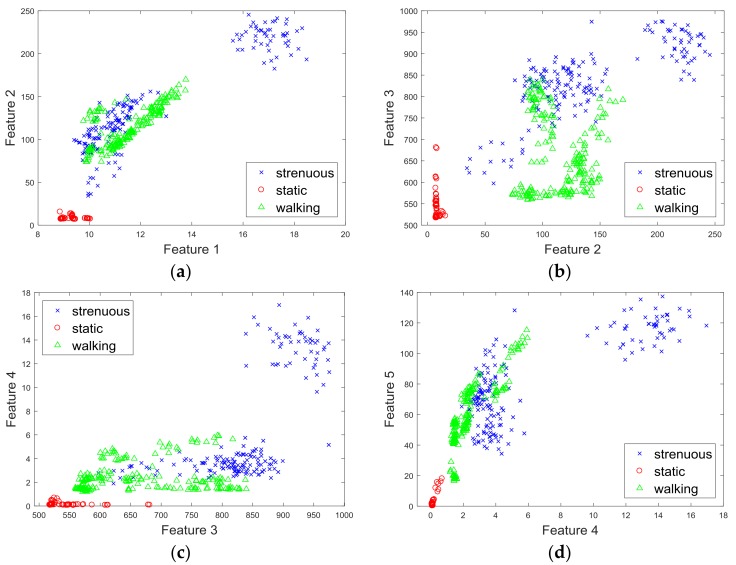
The pairwise scatter plots of the features: (**a**) Mean_1 versus Mean_2; (**b**) Mean_2 versus Mean_PPG; (**c**) Mean_PPG versus Variance_1, and (**d**) Variance_1 versus Variance_2.

After data preprocessing, we needed to extract representative features for activity recognition. According to the experimental observation, the amplitude of the PPG signal response to exercise was distinctly higher than that in the stationary state. Therefore, we measured the average amplitude of the peaks as a distinct feature. In addition, in order to eliminate the effects of sensor orientation, we added a magnitude vector to extract features, e.g., $\sqrt{x^{2}+y^{2}+z^{2}}$. Finally, we used five features to recognize the activities. Table 1 shows the chosen features, and Figure 5 shows the pairwise scatter plots of the features by using 450 samples from 10 different users. As expected, different features were easily distinguishable. Also, the numerical range of the raw PPG signal was around 500, and, thus, almost 20 times larger than the motion data. Thus, we scaled these features between −1 and 1 using Equation (1).

(1) $$\mathrm{scaled}\text{\_}\mathrm{feature}=\;-1+2\times \frac{data-\min(data)}{\max(data)-\min(data)}$$

#### 4.2.2. Classifier Implementation

According to the dataset, the activity recognition approach referred to a three-class classification problem (walking, sitting, and strenuous movements including trotting and ascending and descending stairs). We considered three common machine learning algorithms which have less computational complexity. We used the sklearn python package for our training and evaluation.

##### Linear Support Vector Machine

Linear SVM is an effective machine learning algorithm for solving multiclass classification. In linear SVM, a data point is considered as a p-dimensional vector. We separated points using p − 1 dimensional hyperplane, which is considered to be the one which maximizes the margin. Also, a linear SVM model was created in a CPU (Central Processing Unit) time which scales linearly with the size of the training data set, so there was no need for expensive computing resources. In our experiments we applied the “l2” norm penalty and squared hinge loss for loss function.

##### Nearest-Neighbor

Nearest-neighbor methods are known as nongeneralizing machine learning methods. Despite their simplicity, they have been successfully used in plenty of classification issues. The principle behind nearest-neighbor is to find a predefined number of training samples closest in distance to the new point and predict the label from these. We set the nearest-neighbor parameter k as 5 and used Euclidean distance metric with equal distance weight.

##### Decision Tree

A decision tree is a flowchart-like structure in which each internal node represents a “test” on an attribute. We used the standard CART algorithm [45] to create the decision tree and spilt nodes based on Gini impurity. A response was obtained by following the decisions in the tree from a root node down to a leaf node.

### 4.3. Authentication Method

#### 4.3.1. Feature Extraction

We depicted features by three feature sets: time-domain features, frequency-domain features, and wavelet-domain features. Table 2 gives details about some effective features in the context of activity authentication.

Time-domain features characterize the motion patterns with respect to time. Common time-domain features include the mean, standard deviation, and correlation, etc. We also applied DTW to measure the similarity between two sequences. Firstly, we selected a standard template which had the minimum sum of DTW distance to other samples from the legal user in the training stage, then, we calculated the DTW distance between the template and a certain sample as a feature for this sample.

Frequency-domain shows how much of the signal lies within each given frequency band over a range of frequencies. For instance, different walking speeds can be reflected by different central frequencies. Thus, we estimated the mean normalized frequency and also used the first-half of the FFT (Fast Fourier Transform) coefficients.

Wavelet transform allowed us to localize the feature in both frequency and time. The Discrete Wavelet Transform (DWT) is widely used especially in nonstationary signal analysis. Chen et al. [46] applied a three-order Daubechies wavelet on the wavelet decomposition to data with five levels. In this paper, the sampling rate of the sensor was about 20 Hz, so we applied a fifth-order Daubechies wavelet to data with decomposition at five levels and calculated the wavelet energy on each level as features.

**Figure 6:**
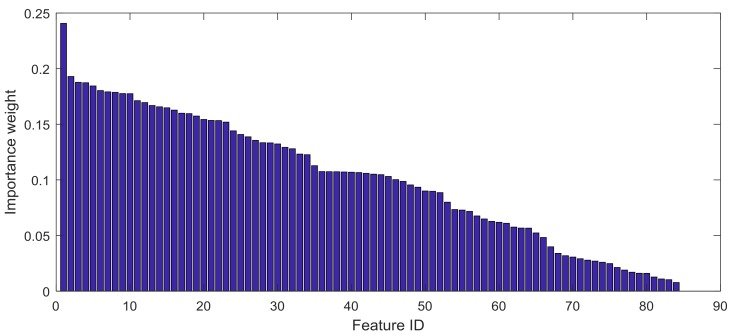
The distribution of importance weights among features.

In order to boost the performance on high-dimensional data and prevent overfitting, we aimed to reduce the dimension of the feature vector by finding a small set of important features which gave a good classification performance. Feature selection algorithms can be roughly grouped into two categories: filter methods and wrapper methods. Filter methods rely on general characteristics of the data to evaluate and to select the feature subsets without involving the chosen learning algorithm. Wrapper methods use the performance of the chosen learning algorithm to evaluate each candidate feature subset. Wrapper methods search for features which have a better fit for the chosen learning algorithm, but they can be significantly slower than filter methods if the learning algorithm takes a long time to run. We used the filter method called ReliefF algorithm [47] to compute the importance of attributes. Figure 6 shows the importance weight from 84 features. In Table 3, we listed the top 10 features which had maximal attribute importance and selected the 90% features with higher importance weight for authentication. All the features are listed in Appendix A.

#### 4.3.2. Classifier Implementation

**Figure 7:**
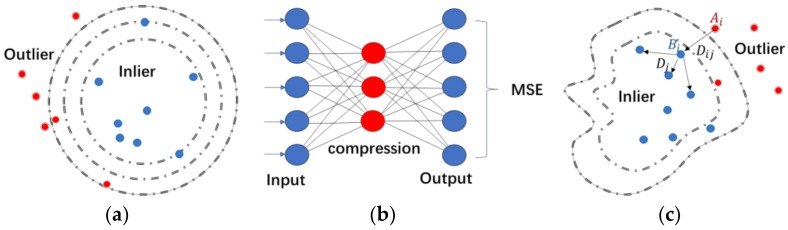
(**a**) The SVM hypersphere, (**b**) The network of autoencoder, (**c**) Topological structure of the k-NN method.

In the certification phase, the classifier decides whether the user is a legitimate user or an imposter. In practical situations, the data sample only comes from the legitimate user, while there are no or few samples from impostors in the training stage. Thus, we considered the authentication task as a one-class classification problem. Various types of one-class classifiers have been designed and applied in different fields. Here we applied three common methods. Figure 7 shows the sketch maps.

##### k-Nearest-Neighbors (k-NN)

A novelty detection method based on Euclidean distance is proposed to address the identity authentication. Specifically, the authentication is divided into two steps: a learning phase and a verification phase. In the learning phase, the Euclidean distance from a training data $A_{i}$ to its nearest neighbor $B_{j}$ is computed and called $D_{ij}$, and then the average distance of the k (k = 10) nearest neighbors for $B_{j}$ is computed and called $D_{j}$. In this way, we could get a vector $D_{ij}/D_{j}$ from all training data. We then set the threshold T equals to the geometric mean of the vector after comparative studies. In the testing phase, we used the same way to calculate the $D_{ml}/D_{l}$ for *m*-th testing data. If $D_{ml}/D_{l}\;$ > threshold, then the m-th testing data is considered from unauthorized user, or else accepted as a legal user.

##### Autoencoder Neural Network

Autoencoder is an unsupervised learning algorithm that applies a reconstruction method to build a one-class classifier. The classifier reproduces the input features at the output layer through minimizing the reconstruction error. The architecture we applied only consists of one hidden layer with 20 neurons. We specified the transfer function as logistic sigmoid function and chose a maximum of 400 training epochs. In the training phase, we used all the training samples to construct the neural network and computed the square error from training sample $A_{i}$ as $E_{i}$, and the standard deviation of the error vector as *std*. Then, we set the threshold according to Equation (2) after comparative studies, where *N* denotes the total number of training samples. In the testing phase, the test data was rejected as an outlier if the reconstruction error was higher than the threshold.

(2) $$\mathrm{Threshold}=\frac{1}{N}\sum_{i=1}^{N}E_{i}+0.2\times std$$

##### One-Class Support Vector Machine

One-class SVM is an unsupervised algorithm that learns a decision function for novelty detection. It separates all the data points from the origin and maximizes the distance from the hyperplane to the origin. We use LIBSVM, an integrated software for SVM, to construct our one-class SVM. The RBF (Radial Basis Function) kernel with parameter gamma and C set to 0.001 and 1.0, respectively, was used. We also assumed that 3% of the observations from the training data were outliers after parameter optimization.

## 5. Result and Analysis

In this section, we present an objective evaluation on the effectiveness of the proposed approach in terms of activity recognition accuracy with various classifiers, authentication accuracy with different motion states, and sensitivity with respect to window size and number of training samples. We also propose an authentication scheme to test the overall performance of our work.

### 5.1. Activity Recognition Results and Analysis 

Firstly, we evaluated the performance of activity recognition by multisensor data. Subjects were instructed to perform the activities including sitting, slow walking, trotting, ascending and descending stairs in turn with a minute of rest between each set. We marked the strenuous movements as class one, sitting as class two, and slow walking as class three. We tested 20 subjects and mingled the data together to examine the performance of the classifiers. The overall amounts of samples were 36,000 from 12,000 samples for each class separately, and the window size was set to 6 s.

**Figure 8:**
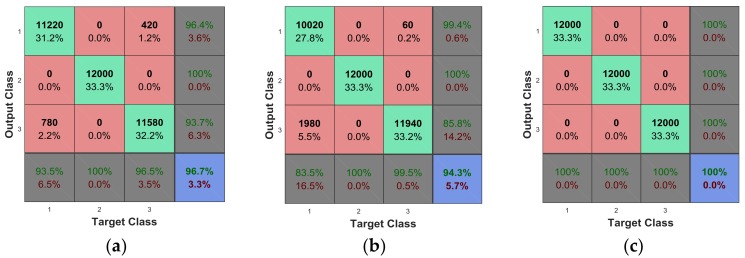
The confusion matrix of the classifier: (**a**) Linear SVM, (**b**) Decision Tree, and (**c**) k-NN.

Figure 8 plots the confusion matrix to show how the currently selected classifier performed in each class. We used 10 cross-validation for training and testing. The k-NN method in Section 4.2.2. showed the best performance with 100% accuracy. The Decision Tree showed the worst performance with 94.3% overall accuracy. Also, it was clearly observed that the recognition accuracy under static conditions was 100% for all the classifiers. On the whole, the results indicate that multisensor data could provide a high recognition precision.

**Figure 9:**
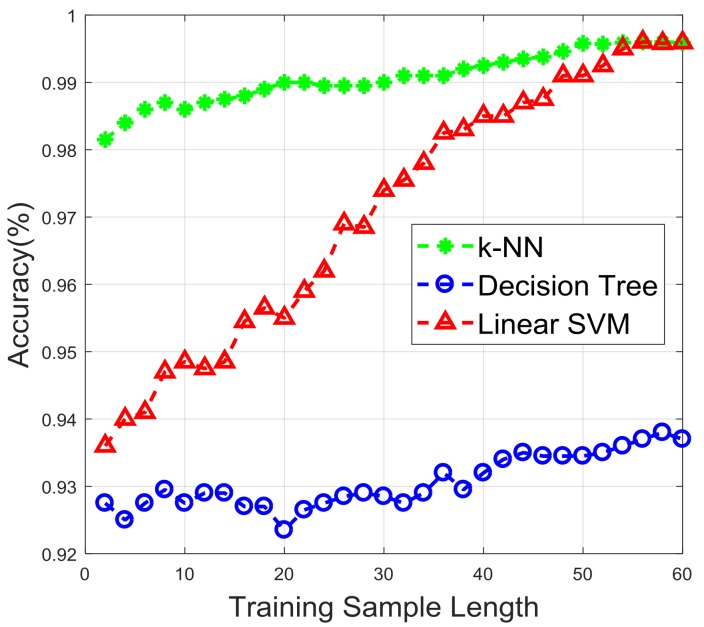
Accuracy against different training sample length.

In practical scenarios, a reasonable number of samples for training is necessary. If the training samples size is too large, the user may feel bored, and the training process will be computationally expensive. On the contrary, if the training samples are insufficient, the prediction accuracy may descend. In order to find a suitable number of training samples, we trained the classifier with different sample sizes. Figure 9 shows the accuracy against variable training sample sizes. We used a training size ranging from 2 to 60 for each class, and tested the performance with 400 samples. It is obvious that the recognition accuracy increased with the number of samples. When the number of training samples reached 45, the growth of accuracy tended to be stable in the k-NN curve. Therefore, in practice, we could determine that a suitable number of training samples was around 45 for each class. Also, the gap between the performance of k-NN and linear SVM gradually narrowed along with the increase of the training sample.

**Figure 10:**
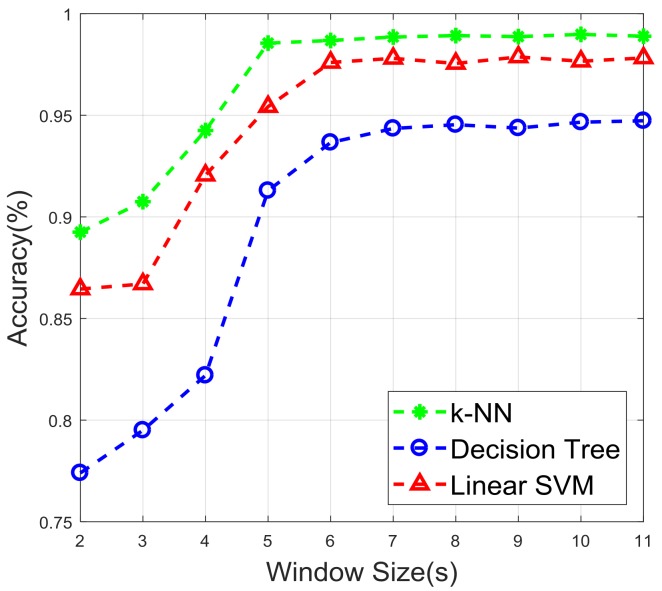
Accuracy against different window sizes.

It has been shown that different window sizes influence the accuracy of a classifier [10] and the recognition speed. The window should contain an appropriate size to differentiate motion states. Figure 10 shows the performance in terms of accuracy according to different window sizes. It can be clearly seen that the classifiers started to provide a fairly optimal performance at the window size 5 s, and the accuracy remained steady after that. Thus, a large window size may be considered unnecessary.

### 5.2. Identity Authentication Results and Analysis

After the process of activity recognition, we could determine the different motion states of the user. We applied an activity specific authentication model and tested the performance under two scenarios: slow walking state and stationary state. We took 40 users in turn as legitimate users and employed 40% of the sample data from the legal user as training data, and all the remaining data from both the legal user and the impostors as testing data. The metrics we evaluated included FAR, FRR, and accuracy rate.

**Figure 11:**
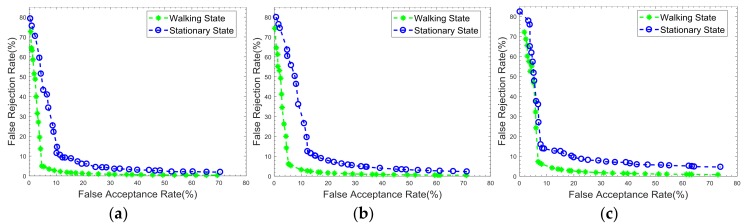
ROC curves under two different scenarios obtained by applying three one-class classification methods: (**a**) SVM, (**b**) Autoencoder, and (**c**) k-NN.

To better illustrate the performance of the classifier, the FAR and FRR were calculated under different decision thresholds. Figure 11 shows the ROC curves of the performance under two authentication states. Table 4 lists the numerical values of FRRs, FARs, and accuracies. The authentication accuracies under two different scenarios were more than 90%. It can also be clearly noticed that the authentication under the walking state showed a better performance compared to the stationary state, which was due to the feature extraction from both motion and physiological data. Specifically, the best performance in the walking state had a FAR of 4.69% and a FRR of 4.95%, while the best performance in the stationary state had a FAR of 10.19% and a FRR of 11.45%. Also, one-class SVM was superior to the other classifiers for the use of kernel function to find the maximum-margin hyperplane. k-NN had a lower performance for the reason that Euclidean distance may have less capacity to distinguish the classes in this case, yet the k-NN had the least computational cost.

**Figure 12:**
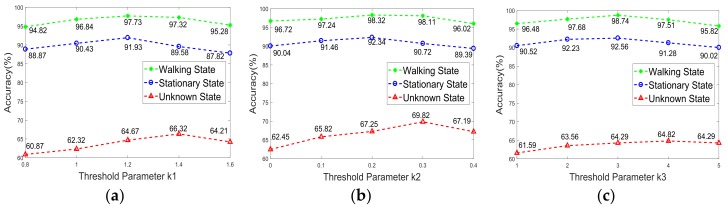
Accuracy under activity-specific case and unknown state case: (**a**) k-NN, (**b**) Autoencoder, and (**c**) SVM.

We considered the case where we used the authentication classifier directly with no prior knowledge of the motion state. In this scenario, the data (PPG and motion data) from both the stationary state and the walking state were mixed together to train the authentication classifier. As shown in Figure 12, we could observe a significant accuracy improvement of the activity-specific authentication classifier with the average improvement around 25%. Hence, it is reasonable to apply an activity recognition classifier before authentication.

### 5.3. System Performance Analysis

**Figure 13:**
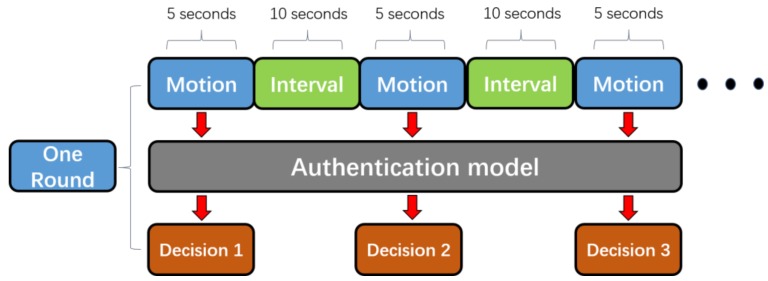
The diagram of the continuous authentication scheme.

We have provided effective methods for both activity recognition and identity authentication. Now, we propose an authentication scheme to combine the activity recognition section with the identity authentication section and we analyze the overall performance. The dataset is available at [43]. Figure 13 shows the diagram of the scheme, and the process is described as follows:

Step 1: We chose 40 participants to train the activity recognition classifier. Each participant was asked to perform five different motions (sitting, slow walking, trotting, ascending and descending stairs) in the shown order, and just one time. Each movement lasted for around 5 s with 10 s break between each of them. In the end, we used 200 labeled samples to train the classifier.

Step 2: We randomly chose a participant as a legitimate user to train the identity authentication classifier. The legal user was asked to achieve 20 sets of sample data on two occasions (sitting, slow walking), respectively. Equally, each set lasted for 5 s with a 10 s rest among them.

Step 3: We considered the rest 39 participants as illegal users. Each of the participant (one legal user and 39 illegal users) performed 20 sets of movement randomly with at least 3 sets for each motion state. Each set lasted at least 10 s. Then, a two-step approach was applied to detect the motion state and the identity.

Step 4: We repeated steps 2 and 3 for 10 rounds and analyzed the overall performance.

We hypothesized that the continuous authentication system would confirm the legal identity of the user if two consecutive certification results were both true. We applied the k-NN method for activity recognition and the one-class SVM for authentication because these classifiers were found to perform better in the previous experiments. Table 5 shows the decision relations in detail. In Table 5, “Miss” means that the activity recognition classifier recognized the strenuous motion state, thus the authentication classifier would not be implemented in this round. “True” indicates the authentication result of the legal user, while “False” indicates the opposite. Decision *i* and Decision *i* + 1 are two continuous authentication processes with intervals of 10 s.

In Table 6, we evaluated the results of the proposed authentication scheme with several representative metrics. The FRR was 0 for all the volunteers which means that all the legal users were identified correctly. The average value of F1-score and FAR were 81.67% and 1.29%, which showed a very promising result.

Our scheme used two consecutive decision to get the final decision for better performance, while increasing the time consumption for certification. The worst case might be the decision sequence “True, False, True, False, …” which could not provide a final decision for a long time. In our experiments, we got the final decision from three single decisions at most. We did not use the average authentication time because the interval time could be set differently under different application scenarios.

## 6. Discussion and Conclusions

This paper proposes a novel method to provide a continuous authentication system using multisensor data both from motion and physiological sensors. We applied three light-weight algorithms to recognize the motion state of users. We then implemented three one-class classification algorithms under two authentication scenarios. Also, we examined the feasibility and usability of the proposed authentication scheme by extensive experiments. The results show that this approach can achieve an average activity recognition accuracy of 99.87%, which indicates that the use of amplitude feature from PPG signals could have a high performance. Also, this approach could achieve a FRR of 4.95% and FAR of 4.69% in the walking state scenario, which proves the great potential of using multisensor data for authentication. In the end, we proposed an authentication scheme and test on 10 volunteers. The results showed an average F1-score of 81.67% and accuracy of 98.5%.

However, there is much work to do in the future. We aim to implement the authentication task through a small-size MCU (Microcontroller Unit) worn by the user which eliminates the cost of a remote computer. More effective feature extraction methods and light-weight algorithms are required to fulfil this demand.

## Figures and Tables

**Table 1:** Features for activity recognition.

Feature	Description
Mean_1 (1) ^1^	Mean value of acceleration magnitude vector
Mean_2 (2)	Mean value of angular velocity magnitude vector
Mean_PPG (3)	The average amplitude from peaks of PPG vector
Variance_1 (4)	Variance value of acceleration magnitude vector
Variance_2 (5)	Variance value of angular velocity magnitude vector

^1^ The number behind the feature represents the order.

**Table 2:** Typical features in different domains. FFT Coefficient $X\left(k\right)=\sum_{n=0}^{N-1}x\left(n\right)W_{N}^{kn}$.

Category	Feature	Description
Time-domain	Mean_1	Mean value of sensor data sequence
	Mean_2	Mean value of local maximum points
	Variance	Variance value of sensor data sequence
	Range	Range value of sensor data sequence
	Kurtosis	Outlier-prone distribution of data sequence
	Skewness	Asymmetry of data sequence around the sample mean
	Moment	Central moment of data sequence
	Interquartile	Difference between the 75th and 25th percentile sequence value
	Cor-coefficient	Linear correlation coefficient between pairs of sequence
	Signal Power	Sum of the absolute squares of data sequence
	DTW distance	Similarity between the data sequence and the template
Frequency-domain	Mean Frequency	Mean normalized frequency of data sequence
	Bandwidth	3-dB bandwidth of power spectral density for data sequence
	Entropy	Shannon entropy of data sequence
Wavelet-domain	FFT Coefficient	Discrete Fourier transform of data sequence
	Wavelet Energy	Wavelet energy of data sequence by Daubechies wavelet

**Table 3:** The top 10 features and their importance weights.

No.	Features	Importance Weight
1	Range of PPG sensor data	0.2407
2	Variance of acceleration magnitude data	0.1929
3	Entropy of PPG sensor data	0.1877
4	Mean frequency of acceleration magnitude data	0.1844
5	50% percentiles of value from acceleration magnitude data	0.1803
6	Mean value of peaks in PPG sensor data	0.1791
7	Variance of angular velocity magnitude data	0.1787
8	Mean absolute deviation of accelerometer data in *x*-axis	0.1776
9	Quantiles of acceleration magnitude data with 0.4 probability	0.1775
10	Geometric mean of acceleration magnitude data	0.1712

**Table 4:** FAR, FRR, and Accuracy performance under two different scenarios using three different classifiers.

Scenario	Parameter	SVM	Autoencoder	k-NN
Walking State	FAR	4.69%	5.29%	6.85%
	FRR	4.95%	6.16%	7.28%
	Accuracy	98.74%	98.32%	97.73%
Stationary State	FAR	10.19%	12.32%	9.82%
	FRR	11.45%	12.47%	15.88%
	Accuracy	92.56%	92.34%	91.93%

**Table 5:** The decision relations of the proposed authentication method.

Round 1	Round 2	Final Decision
True	True	True
False	False	False
True	False	Undecided
False	True	Undecided
Miss	True	Undecided
True	Miss	Undecided
Miss	False	Undecided
False	Miss	Undecided
Miss	Miss	Undecided

**Table 6:** Four metrics among different volunteers.

Volunteer	Accuracy	FRR	FAR	F1-Score
1	97.50%	0	2.56%	66.67%
2	95.00%	0	2.63%	50.00%
3	100%	0	0	100%
4	97.5%	0	2.56%	66.67%
5	100%	0	0	100%
6	97.5%	0	2.56%	66.67%
7	100%	0	0	100%
8	100%	0	0	100%
9	97.5%	0	2.56%	66.67%
10	100%	0	0	100%
Average	98.5%	0	1.29%	81.67%

## Appendix A

The features for training and testing the authentication classifiers are listed in Table A1.

**Table A1:** The list of the features we extracted before using the ReliefF algorithm.

Number	Feature
1	The geomean of acceleration magnitude data
2	The geomean of angular velocity magnitude data
3	The geomean of PPG signal data
4	The average value of local maximum of acceleration magnitude data
5	The average value of local maximum of angular velocity magnitude data
6	The average value of local maximum of PPG signal data
7	The standard deviation of acceleration data in *x*-axis
8	The standard deviation of angular velocity data in *x*-axis
9	The standard deviation of PPG signal data
10	The kurtosis of acceleration data in *x*-axis
11	The kurtosis of angular velocity data in *x*-axis
12	The kurtosis of PPG signal data
13	The skewness of acceleration data in *x*-axis
14	The skewness of angular velocity data in *x*-axis
15	The skewness of PPG signal data in *x*-axis
16	4-order central moment of acceleration magnitude data
17	4-order central moment of angular velocity magnitude data
18	4-order central moment of PPG signal data
19	The range of acceleration magnitude data
20	The range of angular velocity magnitude data
21	The range of PPG signal data
22	The interquartile range of acceleration magnitude data
23	The interquartile range of angular velocity magnitude data
24	The interquartile range of PPG signal data
25	The mean absolute deviation of acceleration magnitude data
26	The mean absolute deviation of angular velocity magnitude data
27	The mean absolute deviation of PPG signal data
28	The 30% percentile value of acceleration magnitude data
29	The 30% percentile value of angular velocity magnitude data
30	The 30% percentile value of PPG signal data
31	The quantiles of acceleration magnitude data for cumulative probability 0.4
32	The quantiles of angular velocity magnitude data for cumulative probability 0.4
33	The quantiles of PPG signal data for cumulative probability 0.4
34	The mean normalized frequency of acceleration data in *x*-axis
35	The mean normalized frequency of angular velocity data in *x*-axis
36	The mean normalized frequency of PPG signal in *x*-axis
37	The average power of acceleration magnitude data
38	The average power of angular velocity acceleration data
39	The average power of PPG signal data
40	The DTW value of acceleration magnitude data
41	The DTW value of angular velocity magnitude data
42	The DTW value of PPG signal data
43	The 3-dB bandwidth of acceleration magnitude data
44	The 3-dB bandwidth of angular velocity magnitude data
45	The 3-dB bandwidth of PPG signal data
46	The root mean square of acceleration magnitude data
47	The root mean square of angular velocity magnitude data
48	The root mean square of PPG signal data
49	The Shannon entropy of acceleration magnitude data
50	The Shannon entropy of angular velocity magnitude data
51	The Shannon entropy of PPG signal data
52–56	Fifth-order Daubechies wavelet energy of acceleration magnitude data
57–61	Fifth-order Daubechies wavelet energy of angular velocity magnitude data
62–66	Fifth-order Daubechies wavelet energy of PPG signal data
67–72	First-half of the FFT coefficients of acceleration data in *x*-axis
73–78	First-half of the FFT coefficients of angular velocity data in *x*-axis
79–84	First-half of the FFT coefficients of PPG signal data in *x*-axis
